# Cinnamon oil downregulates virulence genes of poultry respiratory bacterial agents and revealed significant bacterial inhibition: An *in vitro* perspective

**DOI:** 10.14202/vetworld.2019.1707-1715

**Published:** 2019-11-04

**Authors:** Ahmed Mohammed Erfan, Sherif Marouf

**Affiliations:** 1 National Laboratory for Veterinary Quality Control on Poultry Production, Animal Health Research Institute, Dokki, Giza, 12618, Egypt; 2 Department of Microbiology, Faculty of Veterinary Medicine, Cairo University, Giza, 12211, Egypt

**Keywords:** bacteria, cinnamon, expression, gene sequence, poultry, respiratory

## Abstract

**Background and Aim:**

Respiratory bacterial agents represent one of the most harmful factors that ordinarily threaten the poultry industry and usually lead to great economic losses. Meanwhile, there is a global demand to avoid the highly emerging antibiotic resistance and antibiotic residues in edible meat. Whereas, the use of alternatives became of great priority, especially for those substances extracted from natural plant origin. The study aimed to evaluate the antibacterial effect of cinnamon oil as a herbal extract on different respiratory bacterial agents.

**Materials and Methods:**

One hundred and fifty biological samples were collected through targeted surveillance for respiratory diseased poultry farms representing three governorates, from which bacterial isolation and identification, DNA sequencing of representative strains were performed. Furtherly, phenotypic and genotypic evaluation of the antibacterial effect of cinnamon oil was performed by minimum inhibitory concentration, agar disk diffusion, and virulence genes expression real-time polymerase chain reaction.

**Results:**

Cinnamon oil gave rise to acceptable degrees of virulence genes downregulation of 0.15, 0.19, 0.37, 0.41, 0.77, and 0.85 for *Staphylococcus aureus sed* gene, *Escherichia coli stx1* gene, *Avibacterium paragallinarum HPG-2* gene, *Pasteurella multocida ptfA* gene*, Mycoplasma gallisepticum Mgc2* gene, and *Ornithobacterium rhinotracheale adk* gene, respectively. Phenotypically, using agar disk diffusion assay and broth microdilution susceptibility, cinnamon oil showed also tolerable results as it stopped the growth of *S. aureus, E. coli*, *P. multocida*, and *A. paragallinarum* with varying zones of inhibition.

**Conclusion:**

The encountered results declared the successful *in vitro* effect of cinnamon oil that recommends its application for living birds for future use as a safe antibacterial in the poultry industry.

## Introduction

Respiratory diseases are a significant component of the overall disease incidence in poultry [1]. In many cases, respiratory disease cases detected in poultry farms may represent a part of a predominant disease with lower input of other biosystems [2]. Different microbial agents can initiate respiratory disease syndromes in poultry [3]. Environmental distress can raise the effect of such pathogens, leading to more serious disease cases [4]. Several disease agents as *Ornithobacterium rhinotracheale*, *Escherichia coli*, *Avibacterium paragallinarum, Pasteurella multocida, Mycoplasma gallisepticum*, and *Bordetella avium* were frequently isolated from poultry suffering from respiratory diseases complexes [5]. One approach implemented to overcome the bacterial resistance mechanisms are the use of plant extracts or essential oil either singly or in combination. It also protects the birds from the possible adverse effects of the chemical antibiotics and moreover, protects the consumers from the harmful effect of the residues of the antibiotics that remain in birds’ meat and organs [6]. The antimicrobial activity of essential oils and their components has been recognized for a very long time. Essential oils are made from a very complex mixture of volatile molecules that are produced by the secondary metabolism of aromatic and medicinal plants and can be obtained by distillation of different parts of plants [7]. Herbs and spices have been used since ancient times, not only as antioxidants and flavoring agents but also for their antimicrobial activity [8]. The essential oils of aromatic plants are commonly used in food preservation and flavoring, such as cinnamon (*Cinnamomum zeylanycum Boiss*), a member of the *Lauraceae* family that grows in Southern Asia. It is well documented that compounds that have phenolic groups were the most effective; thus, the oils of cinnamon, thyme, and rosemary have been found to be most effective against foodborne microorganisms [9]. The antimicrobial effect of cinnamon essential oil against various bacteria such as *E. coli, Pseudomonas aeruginosa, Enterococcus faecalis, Staphylococcus aureus, Salmonella* spp., and *Vibrio parahaemolyticus* has been reported [10]. The prevalent active compound that was usually reported in cinnamon oil is cinnamaldehyde [11]. A past study [12] summarized the antibacterial modes of action of cinnamaldehyde in the following points: Loss of pool metabolites, inhibition of active transport, and disruption of DNA, RNA, proteins, lipids, and polysaccharides synthesis, inhibition of the proton motive force, respiratory chain, electron transfer, and substrate oxidation. Another mode of action of volatile oils is their hydrophobicity, which aids them to disturb the lipid bilayer of the cell membrane, resulting in increased permeability to protons [13]. The overall leakage from bacterial cells and the exit of critical molecules leads to bacterial cell death [11].

The significance of the study becomes clear as there are many methods for studying antibacterial mechanisms from which testing bacterial virulence genes expression on mRNA by real-time polymerase chain reaction (PCR) is a very promising tool. To determine mRNA gene expression, quantitative real-time reverse transcriptase PCR is one of the most commonly used molecular techniques as it usually shows high specificity, sensitivity, reproducibility, and a wide dynamic range [14].

The study aimed to assess the possible antibacterial effect of cinnamon essential oil against surveyed bacterial agents incriminated in poultry suffering from respiratory manifestation and to determine through minimum inhibitory concentration (MIC) and also through studying the expression of specific genes by real-time PCR.

## Materials and Methods

### Ethical approval

The current study was approved by the Ethical Committee for live birds sampling at the Animal Health Research Institute, Egypt (License No. AHRI 42102017), according to local Egyptian laws.

### Samples collection

During 3 months (October 2017-December 2017), 150 samples (30 infraorbital sinus exudates, 50 trachea, 20 air sacs, and 50 lung tissues) were collected from poultry farms showing respiratory manifestations in three Egyptian Governorates representing middle, lower, and upper Egypt (Giza, El-Kalyobia, and Beni Suef, respectively). Samples were aseptically collected, transported to the lab by the research team.

### Microbiological analysis

All samples were pre-enriched into broth media; brain heart infusion (BHI) broth (Oxoid, Basingstoke, Hampshire, England, UK) or *Mycoplasma* broth (Oxoid, Basingstoke, Hampshire, England, UK) in case of *Mycoplasma* detection. A loopful was streaked into different microbiological media including 5% sheep blood agar media (Columbia Blood Agar base; Oxoid, Basingstoke, Hampshire, England, UK), MacConkey agar (Thermo Fisher Scientific, GmbH, Germany), Mannitol Salt Agar (HiMedia Laboratories, Mumbai), and *Mycoplasma* agar media (Oxoid, Basingstoke, Hampshire, England, UK). The inoculated plates were incubated for 48 h at 37°C in aerobic or anaerobic conditions except for *Mycoplasma* species where the incubation period extended up to 14 days under microaerophilic condition. Pure cultures were identified biochemically by conventional methods [15-17].

### Molecular identification

#### PCR

All biochemically identified isolates were DNA extracted using QIAamp DNA Mini kit (Qiagen, GmbH, Germany), then genetically identified by PCR using specific primers supplied from Metabion (Germany) and Bio Basic (Canada). NanoDrop ND-1000 spectrophotometer (NanoDrop, USA) was used to check the quantity and quality of the purified DNA. The sequences and cycling conditions of the different used PCR primers are listed in Table-1 [18-23]. PCR reaction (25 μl) contained 12.5 μl of EmeraldAmp GT PCR Master Mix (Takara, Japan), 1 μl of 20 pmol concentration of each primer, 4.5 μl of water, and 6 μl of the DNA template. PCR reactions were performed in Applied Biosystems 2720 Thermal Cycler. Each PCR product was loaded in a separate well in 1.5% agarose gel, then photographed and analyzed using a gel documentation system (Alpha Innotech, Biometra, Germany) through its computer software (BioDocAnalyze 2.64.8.1).

**Table-1:** Sequences and cycling conditions of the different used PCR primers for the amplification of different bacterial isolates.

Bacterial agent	Gene	Primer sequencing	Amplified segment	Amplification (35 cycles)			References

				Secondary denaturation	Annealing	Extension	
*S. aureus*	*clfA*	GCAAAATCCAGCACAAC AGGAAACGA	638 bp	94°C 1 min	55°C 1 min	72°C 1 min	[18]
		CTTGATCTCCAGCCATA ATTGGTGG					
*A. paragallinarum*	*HPG-2*	TGAGGGTAGTCTTGCA CGCGAAT	500 bp	94°C 30 s	63°C 40 s	72°C 40 s	[19]
		CAAGGTATCGATCGTC TCTCTACT					
*M. gallisepticum*	*Mgc2*	CGCAATTTGGTCCTAA TCCCCAACA	300 bp	94°C 30 s	60°C 30 s	72°C 30 s	[20]
		TAAACCCACCTCCAGC TTTATTTCC					
*E. coli*	*phoA*	CGATTCTGGAAATGGCAAAAG	720 bp	94°C 30 s	55°C 45 s	72°C 45 s	[21]
		CGTGATCAGCGGTGA CTATGAC					
*P. multocida*	*Kmt1*	ATCCGCTATTTACCCAGTGG	460 bp	94°C 30 s	55°C 40 s	72°C 45 s	[22]
		GCTGTAAACGAACTCGCCAC					
*O. rhinotracheale*	*16S rRNA*	GAGAATTAATTTACG GATTAAG	784 bp	94°C	58°C	72°C	[23]
		TTCGCTTGGTCTCCGAAGAT		30 s	40 s	50 s	

*S. aureus*=*Staphylococcus aureus*, *E. coli*=*Escherichia coli, A. paragallinarum*=*Avibacterium paragallinarum*, *P. multocida*=*Pasteurella multocida, M. gallisepticum*=*Mycoplasma gallisepticum, O. rhinotracheale*=*Ornithobacterium rhinotracheale,* PCR*=*Polymerase chain reaction

### Differential gene expression real-time PCR

#### Bacterial RNA extraction

Before RNA purification from bacterial harvests, 1 ml of RNAprotect Bacteria Reagent (Qiagen, Germany, GmbH) was mixed with 0.5 ml of the fresh bacterial broth, kept for 5 min at room temperature to prevent bacterial RNA degradation. Then, 200 µl of Tris EDTA buffer containing 1 mg/ml Lysozyme (Thermo Fisher Scientific, GmbH, Germany) was added to pelleted bacteria. Bacterial RNA extraction was performed following the “Enzymatic Lysis” procedure of QIAamp RNeasy Mini kit (Qiagen, Germany, GmbH). During RNA extraction, on-column DNase digestion was done to remove residual DNA.

### SYBR green real-time PCR

PCR reaction was applied in a Stratagene MX3005P real-time PCR machine where the specific primers were utilized in a one-step 25 µl reaction comprising 12.5 µl of the 2× QuantiTect SYBR Green PCR Master Mix (Qiagen, Germany, GmbH), 0.25 µl of RevertAid Reverse Transcriptase (200 U/µL) (Thermo Fisher Scientific, GmbH, Germany), 0.5 µl of different primer (20 pmol conc.), 8.25 µl of PCR grade water, and 3 µl of purified RNA.

The relative expression of each virulence gene was normalized using the related bacterial housekeeping gene. Relative quantitation of gene expression on the RNA templates of the different samples was estimated, the CT value of each sample was compared with that of the control untreated sample through the ΔΔCt method, and samples were tested in triplicates [24]. Primers sequences for reference housekeeping and target genes for each microbial agent are listed in Table-2 [19,20,22,23,25-32].

**Table-2:** Sequences and cycling conditions of the different used PCR primers for SYBR green real-time PCR gene expression studies.

Bacterial agent	Gene	Primer sequencing	Amplification (35 cycles)			References

			Secondary denaturation	Annealing	Extension	
*S. aureus*	*Sed*	CCAATAATAGGAGAAAATAA AAG	94°C 30 s	57°C 30 s	72°C 30 s	[25]
		ATTGGTATTTTTTTTCGTTC				
	*16S rRNA*	CAACGAGCGCAACCCTTAAG	94°C 30 s	57°C 30 s	72°C 30 s	[26]
		TTTGTCACCGGCAGTCAACTT				
*A. paragallinarum*	*HPG-2*	TGAGGGTAGTCTTGCACGC GAAT	94°C 30 s	63°C 40 s	72°C 40 s	[19]
		CAAGGTATCGATCGTCTCTC TACT				
	*gyrA*	AGTGAGCGTAACGGCAAAGT	94°C 30 s	58°C 30 s	72°C 30 s	[27]
		ATGTCCGATTCTTCGTCGTC				
*M. gallisepticum*	*Mgc2*	CGCAATTTGGTCCTAATCCCC AACA	94°C 30 s	60°C 30 s	72°C 30 s	[20]
		TAAACCCACCTCCAGCTTTAT TTCC				
	*16S rRNA*	GAGCTAATCTGTAAAGTTGGTC	94°C 30 s	55°C 30 s	72°C 30 s	[28]
		GCTTCCTTGCGGTTAGCAAC				
*E. coli*	*Stx1*	ATG TCA GAG GGA TAG ATC CA	94°C 30 s	56°C 30 s	72°C 30 s	[29]
		TAT AGC TAC TGT CAC CAG ACA AT				
	*16S rRNA*	GCTGACGAGTGGCGGACGGG	94°C 30 s	55°C 30 s	72°C 30 s	[30]
		TAGGAGTCTGGACCGTGTCT				
*P. multocida*	*ptfA*	TGTGGAATTCAGCATTTTAGTG TGTC	94°C 30 s	55°C 40 s	72°C 45 s	[31]
		TCATGAATTCTTATGCGCAAAA TCCTGCTGG				
	*Kmt1*	ATCCGCTATTTACCCAGTGG	94°C 30 s	55°C 40 s	72°C 45 s	[22]
		GCTGTAAACGAACTCGCCAC				
*O. rhinotracheale*	*adk*	GGCAGTGGAAAAGGAACTCA	94°C 30 s	52°C 30 s	72°C 30 s	[32]
		TCTAAACTTCCTTCGCCGTTT				
	*16S rRNA*	GAGAATTAATTTACGGATTAAG	94°C	58°C	72°C	[23]
		TTCGCTTGGTCTCCGAAGAT	30 s	40 s	50 s	

*S. aureus*=*Staphylococcus aureus*, *E. coli*=*Escherichia coli, A. paragallinarum*=*Avibacterium paragallinarum*, *P. multocida*=*Pasteurella multocida, M. gallisepticum*=*Mycoplasma gallisepticum, O. rhinotracheale*=*Ornithobacterium rhinotracheale,* PCR*=*Polymerase chain reaction

### DNA sequencing

Partial sequencing of different confirmatory genes for different tested bacterial pathogens (*S. aureus clfA*, *A. paragallinarum HPG-2*, *M. gallisepticummgc2*, *E. coli phoA*, *P. multocida kmt1*, and *O. rhinotracheale 16S rRNA*) was performed using specific primers. QIAquick PCR product extraction kit (Qiagen, Valencia) was used to purify PCR products. Bigdye Terminator V3.1 Cycle Sequencing Kit (PerkinElmer) was used to perform sequencing reactions, and then it was purified using Centri-Sep Spin Column. DNA sequences were generated using Applied Biosystems 3130 Genetic Analyzer (HITACHI, Japan). Sequence identity to GenBank accessions was established through BLAST^®^ analysis (Basic Local Alignment Search Tool) [33]. The sequence identities were determined by the MegAlign module of Lasergene DNAStar [34], and phylogenetic analyses were done using maximum likelihood, neighbor-joining, and maximum parsimony in MEGA6 [35].

### Antimicrobial effect of cinnamon essential oil

Commercial essential oil (10.000 μg/mL) was obtained from National Research Center, Dokki, Giza. The antimicrobial activity of cinnamon essential oil was determined using agar disk diffusion assay and broth microdilution susceptibility test against different bacterial isolates [10].

### Agar disk diffusion assay

One hundred microliters of bacterial suspensions (1.5×10^8^ CFU/mL) were spread on Mueller-Hinton agar plates and then filter paper disks (6 mm in diameter) were impregnated with 10 μL of the cinnamon essential oil (5000 μg/mL). The plates were incubated aerobically or anaerobically at 37°C for 24-48 h according to tested bacteria. The diameters of the inhibition zones were measured in mm. All experiments were performed in triplicates [10].

### Microdilution susceptibility test

According to a previous study [10], two-fold serial dilutions of the cinnamon oil were diluted in BHI broth. The following concentrations of essential oil (5000-156.25 μg/mL with two folds decreasing concentrations in between) were used. Twenty microliters of the inoculum were added to each well of a 96-well microplate containing 160 μL BHI broth. Twenty microliters from the stock solutions of cinnamon oil were added into each well; the last well in each raw contained 180 μL of broth and 20 μL of the inoculum without any cinnamon oil as a negative control. The microplates were incubated in standard conditions (according to used microorganisms). Thereafter, the absorbance (A560) for the plates was read by microplate reader spectrophotometry (BioTek Instruments Inc., USA). The MIC was defined as the lowest concentration of the antimicrobial (cinnamon oil) at which the microorganism did not show visible growth. All experiments were performed in triplicates.

### Statistical analysis

The data were analyzed using SPSS version 19 software (SPSS Inc., Chicago, IL), and one-way ANOVA was performed. P=0.05 was the upper limit to determine the statistically significant results. The results were expressed as mean±standard deviations of triplicate measurements.

## Results

### Incidence of bacteria isolated from the respiratory system

As shown in Table-3, phenotypic characterization (growth characteristics, colony morphology, and biochemical reactions) revealed 48 bacterial isolates (related to six microbial agents) with overall incidence of 32%. Identified microbial agents were *E. coli, S. aureus, O. rhinotracheale, P. multocida, A. paragallinarum*, and *M. gallisepticum*. The incidence of those isolates from each governorate and each sample is presented in Table-3. The three governorates showed variable incidences for bacterial isolation; 15.3%, 9.3%, and 7.3% for Giza, Beni Suef, and El-Kalyobia, respectively.

**Table-3:** The incidence of bacteria isolated from different samples from three governorates.

Governorate	Types of samples	No.	*S. aureus*		*O. rhinotracheale*		*A. paragallinarum*		*M. gallisepticum*		*E. coli*		*P. multocida*	
					
			No.	%	No.	%	No.	%	No.	%	No.	%	No.	%
Giza	Exudate	15	4	26.6	0	0	1	6.6	0	0	0	0	1	6.6
	Trachea	20	0	0	2	10	0	0	5	25	0	0	0	0
	Air sac	8	0	0	0	0	0	0	2	25	0	0	0	0
	Lung fragment	20	0	0	0	0	1	5	5	25	2	10	0	0
El-Kalyobia	Exudate	5	1	20	0	0	0	0	0	0	0	0	0	0
	Trachea	15	0	0	0	0	0	0	0	0	0	0	0	0
	Air sac	5	0	0	0	0	0	0	2	40	0	0	0	0
	Lung fragment	15	0	0	0	0	1	6.6	5	33.3	2	13.3	0	0
Beni Suef	Exudate	10	1	10	0	0	0	0	0	0	0	0	3	30
	Trachea	15	0	0	0	0	0	0	1	6.6	0	0	0	0
	Air sac	7	0	0	0	0	0	0	2	28.5	1	14.2	0	0
	Lung fragment	15	0	0	0	0	2	13.3	3	20	1	6.6	0	0
Total		150	6	4	2	1.3	5	3.3	25	16.7	6	4	4	2.6

*S. aureus*=*Staphylococcus aureus*, *E. coli*=*Escherichia coli, A. paragallinarum*=*Avibacterium paragallinarum*, *P. multocida*=*Pasteurella multocida, M. gallisepticum*=*Mycoplasma gallisepticum, O. rhinotracheale*=*Ornithobacterium rhinotracheale*

*M. gallisepticum* showed the highest isolation incidence (16.7%); while only two *O. Rhinotracheale* isolates were obtained with an incidence of 1.3%.

**Figure-1:**
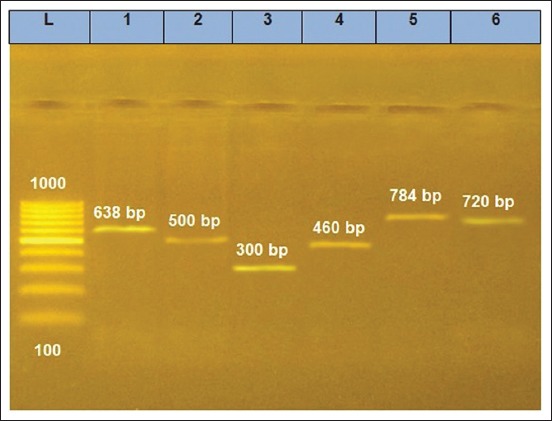
Electrophoresis results for polymerase chain reaction products related to different microbial agents. L, Generuler 100 bp Ladder (100-1000 bp ladder - SM0241, Thermo Fisher Scientific, GmbH, Germany). 1-6 lanes: (1) *Staphylococcus aureus*, (2) *Avibacterium paragallinarum*, (3) *Mycoplasma gallisepticum*, (4) *Pasteurella multocida*, (5) *Ornithobacterium rhinotracheale*, (6) *Escherichia coli*.

PCR was able to detect the different screening genes for the six different microbial agents. Representative bands for the screening genes are shown in Figure-1. Furthermore, PCR proved the virulence of the isolated microbial agents as all of isolated microbial agents showed related virulence genes; such genes were the key factors for the evaluation of cinnamon oil effect.

### DNA sequence

DNA sequences were generated for the different microbial genes; GenBank accessions for the sequenced amplicons were obtained. The identity percentage between the sequenced strains related to the same microbial agent is shown in Table-4.

**Table-4:** Nucleotide identity matrices for the different bacterial isolates.

Bacterial agent	GenBank accessions	Nucleotide identity among Egyptian strains (%)
*S. aureus*	MG821495-MG821497	99.7-99.8
*A. paragallinarum*	MG821492-MG821494	100
*M. gallisepticum*	MG820791-MG820793	99.3-100
*E. coli*	MG821498-MG821500	100
*P. multocida*	MG821501-MG821503	98.9-100
*O. rhinotracheale*	MG773129-MG773130	99.9

*S. aureus*=*Staphylococcus aureus*, *E. coli*=*Escherichia coli, A. paragallinarum*=*Avibacterium paragallinarum*, *P. multocida*=*Pasteurella multocida, M. gallisepticum*=*Mycoplasma gallisepticum, O. rhinotracheale*=*Ornithobacterium rhinotracheale*

The sequenced strains among the same microbial agents showed very close identity percentages to each other (ranged from 98.9% to 100%). On the other hand, each of the sequenced strains showed very high identity to related pathogenic strains of the same microbial species (*A. paragallinarum* Modesto strain, GenBank accession DQ132874.1), (*S. aureus* R1/bov/2015 strain, GenBank accession KX181853.1), (*E. coli* O180: H26 strain EC-107, GenBank accession CP043217.1), (*M. gallisepticum* strain UHP1, GenBank accession AY556297.1), (*O. rhinotracheale* strain ESV-301, GenBank accession KY809801.1), and (*P. multocida* strain Omsk-13, GenBank accession KP212389.1).

### Determination of virulence genes expression by real-time PCR

**Figure-2:**
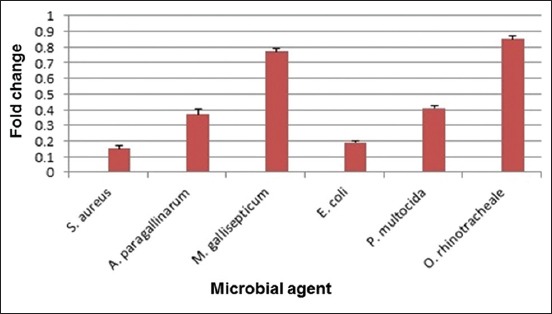
Effect of cinnamon oil on the virulence gene expression of different bacterial agents. Error bars are expressed for mean standard deviations.

Relative quantitation of virulence genes expression by real-time PCR revealed variable degrees of virulence genes downregulation on microbial RNA in relation to the untreated strains (0.15 for *S. aureus sed* gene, 0.19 for *E. coli stx1* gene, 0.37 for *A. paragallinarum HPG-2* gene, 0.41 for *P. multocida ptfA* gene, 0.77 for *M. gallisepticum mgc2* gene, and 0.85 for *O. rhinotracheale adk* gene). The results are shown in Figure-2.

### Antimicrobial activity of cinnamon oil against bacterial isolates

The antimicrobial activity of cinnamon oil was determined using agar disk diffusion and microdilution susceptibility test against the isolated microbial agents.

The results of agar disk diffusion assay for cinnamon oil (p<0.05) revealed significant effect and proved that cinnamon oil could stop the growth of *S. aureus, E. coli*, *P. multocida, and A. paragallinarum*. The inhibition zones were 20.7±0.2 mm, 25.5±0.4 mm, 21.5±0.3 mm, and 27.5±0.6 mm, respectively. However, *O. rhinotracheale and M. gallisepticum* showed very small inhibition zones **(**5±0.6 mm and 3.5±0.2 mm, respectively).

The dose/response or MIC varied according to the microorganism (2500 μg/ml for *S. aureus*, 1250 μg/ml for *E. coli*, 625 μg/ml for both *P. multocida*, and *A. paragallinarum*). However, *O. rhinotracheale* and *M. gallisepticum* were able to grow in all cinnamon oil dilutions.

## Discussion

Respiratory diseases represent a great threat to the poultry industry worldwide. In some cases, such as infectious coryza or infectious laryngotracheitis, the disease may be limited to the respiratory system, at least initially [2]. On the other hand, mixed bacterial infections are significant, and the synergistic role between different pathogenic agents may exist. One hundred and fifty samples were studied for the detection of bacterial respiratory disease agents in poultry farms of Giza, El-Kalyobia, and Beni Suef Governorates. Several microorganisms of the genuses *Pasteurella* (*P. multocida, P. gallinarum, P. haemolytica*, and *P. anatipestifer*), *Bordetella* (*B. avium*), and *Avibacterium* (*A. paragallinarum*) were involved in respiratory diseases complex. *E. coli* associated with respiratory infection in chickens has also been reported [36]. *O. rhinotracheale* has recently been identified as a pathogen causing respiratory tract infections in poultry and other birds [37]. The study revealed 48 bacterial isolates with an incidence of 32% that were identified as *S. aureus, E. coli, O. rhinotracheale, P. multocida, A. paragallinarum*, and *M. gallisepticum*. The incidence of those isolates from each governorate and each sample is analyzed in Table-3. Sequenced strains within the same spp. showed a very close identity to each other, as shown in Table-4.

The low MIC value and encountered inhibition zones revealed by disk diffusion in this study revealed that cinnamon essential oil had antibacterial effect (partial bioactivity) against *S. aureus, E. coli, P. multocida*, and *A. paragallinarum* [8]. Such antimicrobial activity of cinnamon essential oil may be due to the presence of a high concentration of cinnamaldehyde [10]. *O. rhinotracheale* and *M. gallisepticum* grew in all dilution of cinnamon oil that reveals that cinnamon oil had no effect on both organisms. The higher resistance of those bacteria to cinnamon oils is possibly due to differential membrane structure of these bacteria.

The isolated microbial agents were proved to be pathogenic as PCR was able to detect specific virulence genes related to different virulence determinants; fimbrial adhesion (*P. multocida ptfA* gene), enterotoxin (*S. aureus Sed* gene and *E. coli stx1* gene), cytadhesin (*M. gallisepticum mgc2* gene), hemagglutinin (*A. paragallinarum HPG-2* gene), and adenylate kinase (*O. Rhinotracheale adk* gene).

Partial DNA sequencing of representative isolates was performed to give some information about the isolated microbial agents and to determine the degree of homology of the tested isolates of the same microbial agents (to avoid the possible differences on response of bacteria to oil due to sequence difference) before performing gene expression real-time PCR study, this was assumed through the very high degree of similarity between the sequenced fragments of the same agent. Furthermore, DNA sequencing confirmed the pathogenicity of the isolated strains through the high identity percentages with the international pathogenic strains.

To estimate the antimicrobial effect of cinnamon oil, relative quantitation real-time PCR of different virulence genes of the isolated bacterial agents were performed, where it showed a very high degree of bacterial virulence genes downregulation that ranged from 0.15 to 0.85 for *E. coli stx1* gene and *O. rhinotracheale adk* gene, respectively. Real-time PCR results agreed highly with those of phenotypic tests as the highest degree for downregulation was encountered for all microbial agents except for *M. gallisepticum* and *O. rhinotracheale* that showed 0.77 and 0.85 downregulation, respectively. The encountered results confirmed the susceptibility of the different isolated bacterial agents to cinnamon oil and supported the results obtained by the disk diffusion method. Although variable degrees of virulence genes downregulation were recorded, this can be accepted as it may be related to variation in virulence gene expression and microbial response differences.

Relative quantitation real-time PCR results were in great concordance with those obtained by MIC and microdilution susceptibility test for the inhibited bacteria even for *M. gallisepticum* and *O. rhinotracheale* as their virulence genes downregulation was 0.77 and 0.85, respectively; while there was no detectable inhibition for both agents when phenotypic tests were performed.

The varied results encountered for the same essential oil on different pathogenic microbial agents may be explained as the range of the essential oil action against bacteria may only inhibit the bacterial growth (bacteriostatic) or act aggressively to decrease the number of bacterial cells (bactericide). The bacteriostatic action is reversible, as the microbial cells may retrieve their reproductive power after it has been neutralized. However, the bactericidal effect is permanent; as bacterial cells are no more able for growth and reproduction [38].

The degree of downregulation recorded for *M. gallisepticum* and *O. rhinotracheale* was the lowest among the tested bacteria. Those degrees were not sufficient to be translated to phenotypic inhibition. The disagreed phenotypic and molecular results may be related to RNA translation failure. This failure may be due to some factors as generation of a nonsense codon in the mRNA by transcriptional error [39], failure of ribosome binding to mRNA, ribosomal frameshifting on mRNA leading to the premature termination of translation or endonucleolytic mRNA cleavage [40].

The antibacterial role of cinnamon oil on different bacteria was previously encountered by many authors who proved its powerful effect as *S. epidermidis icaA* gene [41], *E. coli* O157:H7 *Stx* gene [42], *S. epidermidis mecA* gene [43], *S. aureus FtsZ* gene [44], and *S. aureus Sea, Sec*, and *See* genes [45]. Such studies recommended cinnamon oil to be used as an alternative for antibacterial medicines.

Different extraction methods have been proved to possess successful antibacterial effect against Gram-negative or Gram-positive microbial agents, as organic solvents extracts [46], stem ethanolic extracts [47], powder hydroethanolic extract [48], combinations with other herbal extracts [49], or hydrodistillation [50]. Those different methods for extracting cinnamon oil can help medicine companies to produce cinnamon oil on commercial levels.

Toxicity is one of the common adverse effects of herbal extracts; due to that phenomenon, cinnamon oil has been recommended to be diluted to 2% in case of oral route application [8].

## Conclusion

The reported results have shed some light on the possible use of cinnamon oil and other essential oils for the control of antibiotic-resistant bacterial infections instead of the widely used antibiotics that usually develop bacterial resistance or cause harmful effects for the birds’ vital organs in addition to the possible residues that remain in the poultry meat. Further testing of different methods of cinnamon oil extraction should be held. Furthermore, before such application of essential oils on farm levels, toxicity studies should be performed to stand on the safe dose to be applicated.

## Recommendations

We recommend studying the antibacterial effect of cinnamon oil *in vivo* in different animals in different dilutions and in different routes.

## Authors’ Contributions

SM designed this study and applied microbiological experiments. AME performed molecular biology tests. Both authors collected samples, drafted, revised the manuscript, analyzed the data, and approved the final manuscript. Both authors read and approved the final manuscript.
